# Correction: The impact of ribosomal interference, codon usage, and exit tunnel interactions on translation elongation rate variation

**DOI:** 10.1371/journal.pgen.1007620

**Published:** 2018-08-24

**Authors:** Khanh Dao Duc, Yun S. Song

In Table 3 of the S1 Data file, the codons in the first column are mislabeled. Please view the correct S1 Data file below where the codons are ordered by ascending mean elongation rate (MER) values. The correct S1 Data file includes MER values in addition to the original statistics.

## Supporting information

S1 Data Data and results for the 850 genes discussed in this paper. (XLSX)
