# Supplementary material for: The impact of ribosomal interference, codon usage, and exit tunnel interactions on translation elongation rate variation
Source: PLoS Genet. 2018 Jan 16;14(1):e1007166. doi: 10.1371/journal.pgen.1007166 (PMC5786338; doi:10.1371/journal.pgen.1007166)
Supplement: S1 Text — (PDF) [file pgen.1007166.s001.pdf]

Supporting Information

The impact of ribosomal interference, codon usage, and exit tunnel interactions on translation elongation rate variation

Khanh Dao Duc[1] and Yun S. Song[1,2,3,*]

1 Computer Science Division, University of California Berkeley, Berkeley, CA 94720
2 Department of Statistics, University of California Berkeley, Berkeley, CA 94720
3 Chan Zuckerberg Biohub, San Francisco, CA 94158, USA

[*] Correspondence should be addressed to: yss@berkeley.edu

S1 Text

1 Description and analysis of the translation model

We here detail the model we used to study the ribosome dynamics along the transcript. The mRNA is modeled as a lattice $[1 : N]$ of N sites, with associated elongation rates $(\lambda_1, \dots, \lambda_N)$ and initiation rate α. A ribosome is modeled as an extended particle of size $\ell = 10$ and we track its A-site position. At any time t, the state of occupancy of the mRNA is given by a vector $\boldsymbol{\tau} = (\tau_1, \dots, \tau_N) \in \{0,1\}^N$, such that τ_i is 1 if position i is occupied by the A-site of a ribosome, and 0 otherwise. During infinitesimal time interval dt, a particle at position i has probability $\lambda_i dt$ to jump to the next site at $i+1$ if position $i+\ell$ is empty. At the boundaries, a particle at position N exits with probability $\lambda_N dt$, and a particle can be injected at position 1 with probability αdt if the first ℓ sites are empty. An initiation event corresponds to when the P-site of a lower ribosomal subunit arrives at the start codon and the upper ribosomal subunit gets assembled, so the first site of the lattice in our mathematical model corresponds to the second codon position and N is equal to the gene length minus 1. In the long time limit, the system is at steady state and the associated marginal density of ribosomes at position i is given by

$$\langle \tau_i \rangle = \left[\sum_{\tau \in \{0,1\}} \tau \mathbb{P}(\tau_i = \tau) \right] = \mathbb{P}(\tau_i = 1). \quad (1)$$

It can also be shown that these densities satisfy the following master equation:

$$0 = \alpha \langle \prod_{j=1}^{\ell} (1 - \tau_j) \rangle - \lambda_1 \langle \tau_1 (1 - \tau_{\ell+1}) \rangle, \quad (2)$$

$$0 = \lambda_i \langle \tau_i (1 - \tau_{i+\ell}) \rangle - \lambda_{i+1} \langle \tau_{i+1} (1 - \tau_{i+\ell+1}) \rangle, \quad \text{for } 1 \le i \le N - \ell - 1, \quad (3)$$

$$0 = \lambda_i \langle \tau_i \rangle - \lambda_{i+1} \langle \tau_{i+1} \rangle \quad \text{for } N - \ell \le i \le N - 1. \quad (4)$$

(See [1] for the master equation of a simpler TASEP model with a constant elongation rate and particle of size one.) For $1 \leq i, j \leq N - \ell - 1$, the densities at position i, j satisfy

$$\lambda_i \langle \tau_i (1 - \tau_{i+\ell}) \rangle = \lambda_j \langle \tau_j (1 - \tau_{j+\ell}) \rangle. \tag{5}$$

We can rewrite this equality as

$$\lambda_i \mathbb{P}(\tau_i = 1, \tau_{i+\ell} = 0) = \lambda_j \mathbb{P}(\tau_j = 1, \tau_{j+\ell} = 0), \tag{6}$$

which yields

$$\lambda_i \mathbb{P}(\tau_i = 1) \mathbb{P}(\tau_{i+\ell} = 0 \mid \tau_i = 1) = \lambda_j \mathbb{P}(\tau_j = 1) \mathbb{P}(\tau_{j+\ell} = 0 \mid \tau_j = 1). \tag{7}$$

If we assume that the interference rate is low at i and j, it means that $\mathbb{P}(\tau_{i+\ell} = 0 \mid \tau_i = 1) \approx 1$ and $\mathbb{P}(\tau_{j+\ell} = 0 \mid \tau_j = 1) \approx 1$. In this case, we then obtain the following relation between the densities

$$\lambda_i \mathbb{P}(\tau_i = 1) \approx \lambda_j \mathbb{P}(\tau_j = 1). \tag{8}$$

In other words, when the interference is low, the density is well approximated, up to a constant (that can be defined via a normalization procedure, see below), by the inverse of its associated rate. We use this approximation below in our rate inference procedure.

2 Simulation of ribosome profiles

We now describe our algorithm for simulating a ribosome profile under our TASEP-based model. We start our simulation at time $t = 0$. At any step of the simulation, we define a vector $(\tau_1, \ldots, \tau_N) \in \{0, 1\}^N$ specifying the ribosome occupancy state of the mRNA (see above), and a list $\Omega = (\omega_1, \ldots, \omega_n)$ of all possible next reactions and their associated times. More precisely, $\omega_k = (\omega_k[1], \omega_k[2]) \in [0 : N] \times \mathbb{R}^+$, where $\omega_k[1] = 0$ corresponds to an initiation event; $\omega_k[1] = i$, where $i \in [1 : N - 1]$, to an elongation event at position i; and $\omega_k[1] = N$ to a termination event at position N. If the current time is t_0, the time of occurrence of the event is $t_0 + \omega_k[2]$. The list Ω is kept ordered by increasing reaction times $\omega_k[2]$, so $\omega_1[2] \leq \omega_2[2] \leq \cdots \leq \omega_n[2]$. Our algorithm can then be summarized as follows.

1. Let $i_0 = \omega_1[1]$.
2. Update times: $t \leftarrow t + \omega_1[2]$ and $\omega_k[2] \leftarrow \omega_k[2] - \omega_1[2]$ for all k.
3. Update the occupancy state :
   (a) If $i_0 = 0$ (initiation), then set $\tau_1 \leftarrow 1$.
   (b) If $i_0 = N$ (termination), then set $\tau_N \leftarrow 0$.
   (c) If $i_0 \in [1 : N - 1]$ (elongation), then set $(\tau_{i_0}, \tau_{i_0+1}) \leftarrow (0, 1)$.
4. Remove the event ω_1 from Ω.
5. Find possible new reactions and sample their associated times :
   (a) If $i_0 = N$ and $\tau_{N-\ell} = 1$, then sample $t_0 \sim \text{Exp}(\lambda_{N-\ell})$ and insert $(N - \ell, t_0)$ into Ω.
   (b) If $i_0 \in [0 : N - 1]$
      i. If $i_0 = \ell$, then sample $t_0 \sim \text{Exp}(\alpha)$ and insert $(0, t_0)$ into Ω.

ii. If $i_0 > \ell$ and $\tau_{i_0-\ell} = 1$, then sample $t_0 \sim \text{Exp}(\lambda_{i_0-\ell})$ and insert $(i_0 - \ell, t_0)$ into Ω.

iii. If $i_0 < N - \ell$ and $\tau_{i_0+\ell+1} = 0$ or if $N - \ell \leq i_0 \leq N - 1$, then sample $t_0 \sim \text{Exp}(\lambda_{i_0+1})$ and insert $(i_0 + 1, t_0)$ into Ω.

6. Go to 1.

To obtain a profile, we first simulate the model for $\sim 10^4$ iterations for burn-in. Then, we further run the simulation algorithm and record the positions of the ribosomes on the mRNA at every fixed time interval of Δt. When we record the positions, we distinguish well-isolated ribosomes from closely-stacked ribosomes (see the **Methods** section), to obtain a profile of detected ribosomes as well as a full profile of all ribosomes. By normalizing the respective sum of ribosome counts by $\frac{T}{\Delta t}$, where T is the total simulation time after burn-in, we obtain the average detected and total density of ribosomes along the mRNA. In practice, we simulated profiles of $N = 15,000$ footprints for genes of length < 500 codons and $N = 30,000$ footprints for genes of length > 500 codons.

3 Inference of initiation and elongation rates

For an experimental profile $p^{\text{exp}} = (p_1^{\text{exp}}, p_2^{\text{exp}}, \ldots, p_N^{\text{exp}})$, where N is the gene length and p_i^{exp} the number of reads detected at position i, and an average detected density D_{exp} (number of detected ribosomes per mRNA), we detail here the procedure to infer the associated elongation rates and initiation rate.

3.1 Naive estimates of elongation rates

To infer the different parameters of the model (namely, the initiation rate and the elongation rates), we first approximate the elongation rate at position i by

$$\lambda_0(i) = \begin{cases} \min\left(\lambda_{\max}, \dfrac{p_{\max}^{\text{exp}}}{p_i^{\text{exp}}}\right), & \text{if } p_i^{\text{exp}} \neq 0, \\ \lambda_{\max}, & \text{else,} \end{cases}$$

where $p_{\max}^{\text{exp}} = \max_i (p_i^{\text{exp}})$ and $\lambda_{\max}$ is a fixed threshold value. In practice, we set $\lambda_{\max}$ to 50, which concerns 0.11% of the sites in our main dataset. As shown in 1, these estimates well approximate (up to a constant determined later on) the true elongation rates when the ribosomes encounter few interference. Using the estimates given by λ_0, we can simulate profiles as described in 2 for any initiation rate value α and obtain the corresponding detected average density $D(\alpha)$. Since D is an increasing function of α, we then estimate the initiation rate by computing $\operatorname{argmin}_\alpha |D_{\text{exp}} - D(\alpha)|$: In practice, we first start our simulations with $\alpha = 0.1$ (the minimal value of the elongation rates is 1). If $D_{\text{exp}} - D(\alpha) > 0$ (respectively, < 0), then we multiply (respectively, divide) α by 2, run the simulations and repeat the procedure until α_1 and α_2 such that D_{exp} is comprised between $D(\alpha_1)$ and $D(\alpha_2)$ (if this is not the case after 5 iterations ($\alpha > 1.6$ or $\alpha < 0.0016$), we stop the procedure and conclude it cannot converge to a fine estimate). Once α_1 and α_2 are found, we use a binary search algorithm (with 5 iterations) to minimize over α the objective function $|D_{\text{exp}} - D(\alpha)|$.

3.2 Detection of sites with significant errors

Using these first estimates, we simulate the associated profile and compare it to p^{exp}. When there is interference at a certain position, the previous estimates may not be accurate and thus lead to

significant errors. To compare the experimental profile p^{exp} to another profile $p = (p_1, p_2, \dots, p_L)$, we define the error at position i as

$$\varepsilon(i) = \left| \frac{p_i^{\text{exp}}}{\sum_k p_k^{\text{exp}}} - \frac{p_i}{\sum_k p_k} \right| .$$

We introduce a significant error threshold, given by

$$\varepsilon_0 = \frac{10 \sum_i \varepsilon(i)}{N} .$$

When the error at a site i_0 is larger than ε_0, we define the complementary set of sites to correct as

$$I_0 = \{ \text{sites } i \in [1:N] \mid i \in [i_0 - 30, i_0 - 10] \text{ and } p_i < p_{\text{th}} \} ,$$

where p_{th} is a fixed threshold value (this choice of set will be explained in the next subsection).

3.3 Refinement step

In the next step of the inference procedure, we correct the elongation rates at i_0 and I_0 to minimize the global error between the corresponding simulated profile and p^{exp}. For a given sequence of rates $\boldsymbol{\lambda} = (\lambda_1, \dots, \lambda_N)$, error site i_0 and corresponding set I_{i_0}, we define the modified sequence

$$\lambda_{\rho_1,\rho_2}^{i_0,I_0}(i) = \begin{cases} \rho_1 \lambda_i, & \text{if } i = i_0, \\ \rho_2 \lambda_i, & \text{if } i \in I_0, \\ \lambda_i, & \text{else,} \end{cases} \tag{9}$$

where $0 < \rho_1, \rho_2$. For such sequence, we can apply the same previous procedure to estimate the initiation rate α_{ρ_1,ρ_2} that fits the experimental average detected density, and get an associated profile p_{ρ_1,ρ_2}. Our goal is then to find the optimal modified sequence minimizing the global error

$$(\hat{\rho}_1, \hat{\rho}_2) = \underset{\rho_1,\rho_2}{\operatorname{argmin}} \left(\varepsilon_{\rho_1,\rho_2} \right) , \tag{10}$$

where

$$\varepsilon_{\rho_1,\rho_2} = \sum_i^N |p_{\rho_1,\rho_2}(i) - p_i^{\text{exp}}| .$$

When there is interference, using naive estimates (obtained for $\rho_1 = \rho_2 = 1$) generates more interference than the true estimates do (the total density observed is only partially explained by the inverse of the true rate). Consequently, the simulated density under these naive rates gets lower than the observed one, as stacked ribosomes get undetected. To increase the simulated density to match the observed one, we thus jointly need to decrease the level of interference generated by high speed traffic upstream (this is why we define I_0 above) and also decrease the speed at the position where the error is detected. To do so, we implemented a double golden-section search to minimize the error for $\rho_1, \rho_2 < 1$: When ρ_1 is fixed, we optimize ρ_2 by golden-section search : We first need to find $a < b < c$ such that $\varepsilon_{\rho_1,b} < \min(\varepsilon_{\rho_1,a}, \varepsilon_{\rho_1,c})$. Starting with $(a, b, c) = (0.8, 0.9, 1)$, the search for a, b and c is done as follows:

1. Compute $(\varepsilon_{\rho_1,a}, \varepsilon_{\rho_1,b}, \varepsilon_{\rho_1,c})$.

2. If $\varepsilon_{\rho_1,b} = \min(\varepsilon_{\rho_1,a}, \varepsilon_{\rho_1,b}, \varepsilon_{\rho_1,c})$, then break.

3. If $\varepsilon_{\rho_1,a} = \min(\varepsilon_{\rho_1,a}, \varepsilon_{\rho_1,b}, \varepsilon_{\rho_1,c})$, then $(a, b, c) \leftarrow (2a - b, a, b)$.

4. If $\varepsilon_{\rho_1,c} = \min(\varepsilon_{\rho_1,a}, \varepsilon_{\rho_1,b}, \varepsilon_{\rho_1,c})$, then $(a, b, c) \leftarrow (b, (b + c)/2, c)$.

5. Go to 1.

When (a, b, c) are determined, we then apply the golden section search algorithm to minimize $\varepsilon_{\rho_1,a}$ over ρ_2. In practice, we run for this optimization step 5 iterations (including the initial search for (a, b, c) and the golden section search). To optimize the error over ρ_1, we embed the optimization of ρ_2 into the same procedure, applied to optimize ρ_1.

If only one error site has been detected, the inference procedure ends after obtaining $\alpha_{\hat{\rho}_1,\hat{\rho}_2}$ and $\lambda^{i_0,I_0}_{\hat{\rho}_1,\hat{\rho}_2}$ as the final estimated initiation and elongation rates. For multiple error sites, we dynamically apply the procedure that we described : We first find the optimal rates for the site closest to the $5'$ end (with the supplementary condition that the modified rates do not introduce significant errors upstream of the original error site). We then update the positions of sites with significant errors, and reiterate the refinement procedure at the closest error site (if there is one) with these new rates, until no error sites are detected downstream of the last error site that was treated.

3.4 Normalization

The elongation and initiation rates obtained after running the inference procedure need to be normalized to get the translation dynamics in appropriate units. To get for each gene the normalization constant, we simulated 10000 ribosomal runs from position 150 and recorded the average time to reach the last codon. After computing the associated average speed v (dividing the length run by ribosomes by the corresponding average time), we normalized the rates by $5.6/v$ to match the experimental observations of Ingolia *et al.*[2], which found a consistent average speed of approximately 5.6 codon/s for each gene.

4 Analysis of the correction procedure

We investigated the main differences between the subsets of genes that required correction during the inference procedure and the ones that did not. First of all, we found larger TEs for genes that required some local correction, with a median TE of 1.11 (mean 1.10), while the other genes had a median TE of 1.04 (mean 1.05). This difference makes sense, as the errors are generated because of interference, which is more likely to occur at high density. Also, we observed that the genes that required correction tend to be slightly longer (median lengths of 435 codons vs. 392 codons), which again makes sense because they contain more sites that can potentially require correction. We further looked at the positions associated with correction (see Figure S3 Fig) and found that 32% (respectively, 53%) were located less than 50 (respectively, 100) codons from the start codon, with a peak corresponding to the top of the ramp pattern (position 40). The $3'$ end was also a large source of correction requirement (in 16% of the genes that needed some correction). Because correction is associated with having regions with interference, the genes present a larger density of footprints in the region where correction is needed, as shown by the metagene profile for the two groups of genes. A potential factor explaining the differences between the two sets of genes could be the initiation rate. However, we found that the initiation rates of genes requiring correction (median = 0.105, mean = 0.098) are on average only slightly larger than that for the genes requiring no correction (median = 0.101, mean = 0.093).

We further looked at the codon context surrounding the inconsistent sites and computed the relative abundance of each codon (see Figure S4 Fig). We first observed some important biases

towards stop codons at the A-site, which is in agreement with Figure S3 Fig. Interestingly, we also found strong biases for codons with the slowest mean elongation rates (CCA, CCG and CGG, red arrows), as computed in Figure 3. We verified (Figure S5 Fig) that the mean elongation rate is not affected by averaging over genes without inconsistent sites. Thus, the fact that these codons are found to be the slowest ones is independent from their presence at inconsistent sites. CGG and to a less extent CGA were also found to be more enriched in the region surrounding the A-site. Overall, these results are consistent with our findings. Because we obtained that the A-site codon was the factor of largest variation of elongation rates, slowest codons should also be the largest factors of interference generating inconsistent sites.

5 Dataset selection

Besides Weinberg *et al.*'s data [3], our choice of datasets for the present study was motivated by comparative analysis of different ribosome profiling datasets (also accessible at riboviz.org [4]). Because of biases at the 5' end inherent to CHX treatment, we first chose to consider only those studies using flash-freeze treatment, leaving Nedialkova, Guydosh, Lareau, Gardin, Pop, Jan, Williams (as named in Weinberg *et al.*'s) [3] datasets. Among them, the best dataset in terms of coverage and correlation of footprint density with A-site codon was that of Williams *et al.*'s [5] (see Weinberg *et al.* Fig. S2 [3], or riboviz.org). Although it is of lesser quality, we also chose to analyse Pop *et al.*'s dataset [6] since it includes RNA-seq data, which allowed us to compute TEs.

6 Comparison of initiation rates inferred from different datasets

We provide here a detailed comparison between the inferred initiation rates from Weinberg *et al.*'s dataset [3] and Pop *et al.*'s dataset. Direct comparison shows a positive correlation of 0.31 (p-value $< 10^{-5}$) (see Figure S10 Fig). Compared to Weinberg *et al.*'s dataset, Pop *et al.*'s ribosome profiling data show a decrease in sequencing depth (which creates more sites with no footprints). More precisely, we found 9.2% of sites with no reads (compared to 0.11% in Weinberg *et al.*'s data). After separating genes into subsets of low and high ratio of missing reads (respectively lower and higher than the median of 8.6%), we found that the initiation rates for genes with less missing reads were better correlated (0.33, p-value $< 10^{-4}$) than for the ones with more missing reads (0.2, p-value $<$ 0.04). This suggests that as the sequencing depth increases, the results of the inference procedure get more consistent.

7 Estimation of drop-off rates

We estimated the drop-off rate using the methods introduced by Sin *et al.* [7]. For each transcript i, we divided its open reading frame (ORF) into bins of 25 codons and counted the number of footprints mapped in each bin j. We normalized this count by the ratio of the total number of footprints in the ORF and the average ribosome density (number of reads per mRNA per 100 codons). From the associated matrix, the drop-off rate is estimated by fitting the average value of each column with Ae^{-QX}, where X is the bin index and A, Q are fitting parameters. The drop-off rate r (per elongation event per codon) is given by $r = 1 - (1 - Q)^{1/25}$.

REFERENCES

1. Derrida B, Domany E, Mukamel D. An exact solution of a one-dimensional asymmetric exclusion model with open boundaries. Journal of Statistical Physics. 1992;69(3-4):667–687.

2. Ingolia NT, Lareau LF, Weissman JS. Ribosome profiling of mouse embryonic stem cells reveals the complexity and dynamics of mammalian proteomes. Cell. 2011;147(4):789–802.

3. Weinberg DE, Shah P, Eichhorn SW, Hussmann JA, Plotkin JB, Bartel DP. Improved ribosome-footprint and mRNA measurements provide insights into dynamics and regulation of yeast translation. Cell Reports. 2016;14(7):1787–1799.

4. Carja O, Xing T, Wallace EWJ, Plotkin JB, Shah P. riboviz: analysis and visualization of ribosome profiling datasets. BMC Bioinformatics. 2017;18(1):461. doi:10.1186/s12859-017-1873-8.

5. Williams CC, Jan CH, Weissman JS. Targeting and plasticity of mitochondrial proteins revealed by proximity-specific ribosome profiling. Science. 2014;346(6210):748–751.

6. Pop C, Rouskin S, Ingolia NT, Han L, Phizicky EM, Weissman JS, et al. Causal signals between codon bias, mRNA structure, and the efficiency of translation and elongation. Molecular Systems Biology. 2014;10(12):770.

7. Sin C, Chiarugi D, Valleriani A. Quantitative assessment of ribosome drop-off in E. coli. Nucleic Acids Research. 2016;44(6):2528–2537.
