# Supplementary figures and images for: The impact of ribosomal interference, codon usage, and exit tunnel interactions on translation elongation rate variation
Source: PLoS Genet. 2018 Jan 16;14(1):e1007166. doi: 10.1371/journal.pgen.1007166 (PMC5786338; doi:10.1371/journal.pgen.1007166)

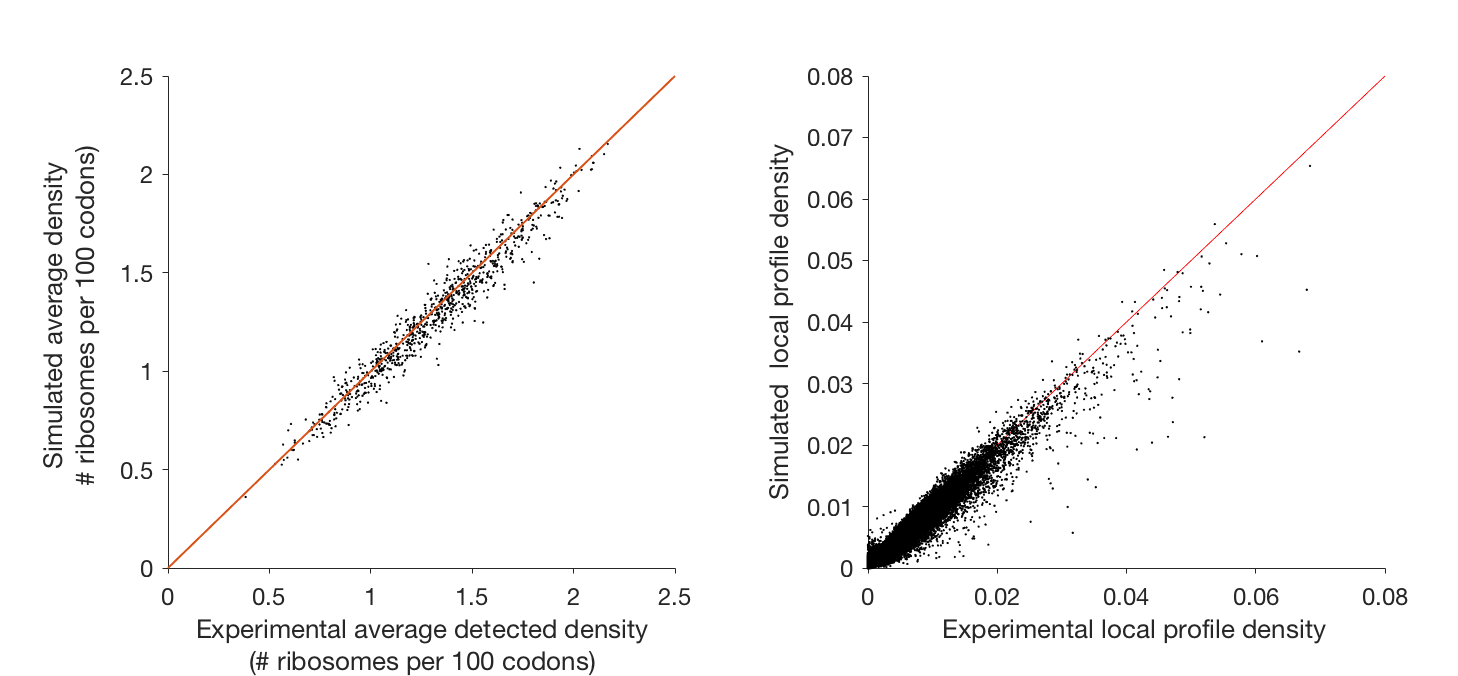

Supplement: S1 Fig — We applied the inference method to a set of 850 genes in S. Cerevisiae (see Materials and Methods) and compared the total (left) and local (right) densities of the original dataset with the ones obtained by simulations of the model with the inferred parameters. The parameters used were pth=10.7λmax=35 and λmax = 50. The simulated and experimental densities were in good agreement, showing a Pearson’s correlation coefficient of 0.986 (p-value < 10−5). The individual profiles obtained by simulations also showed good agreement with the experiments, with Pearson’s correlation coefficient of 0.975 (p-value < 10−5). (TIF) [file pgen.1007166.s002.tif]

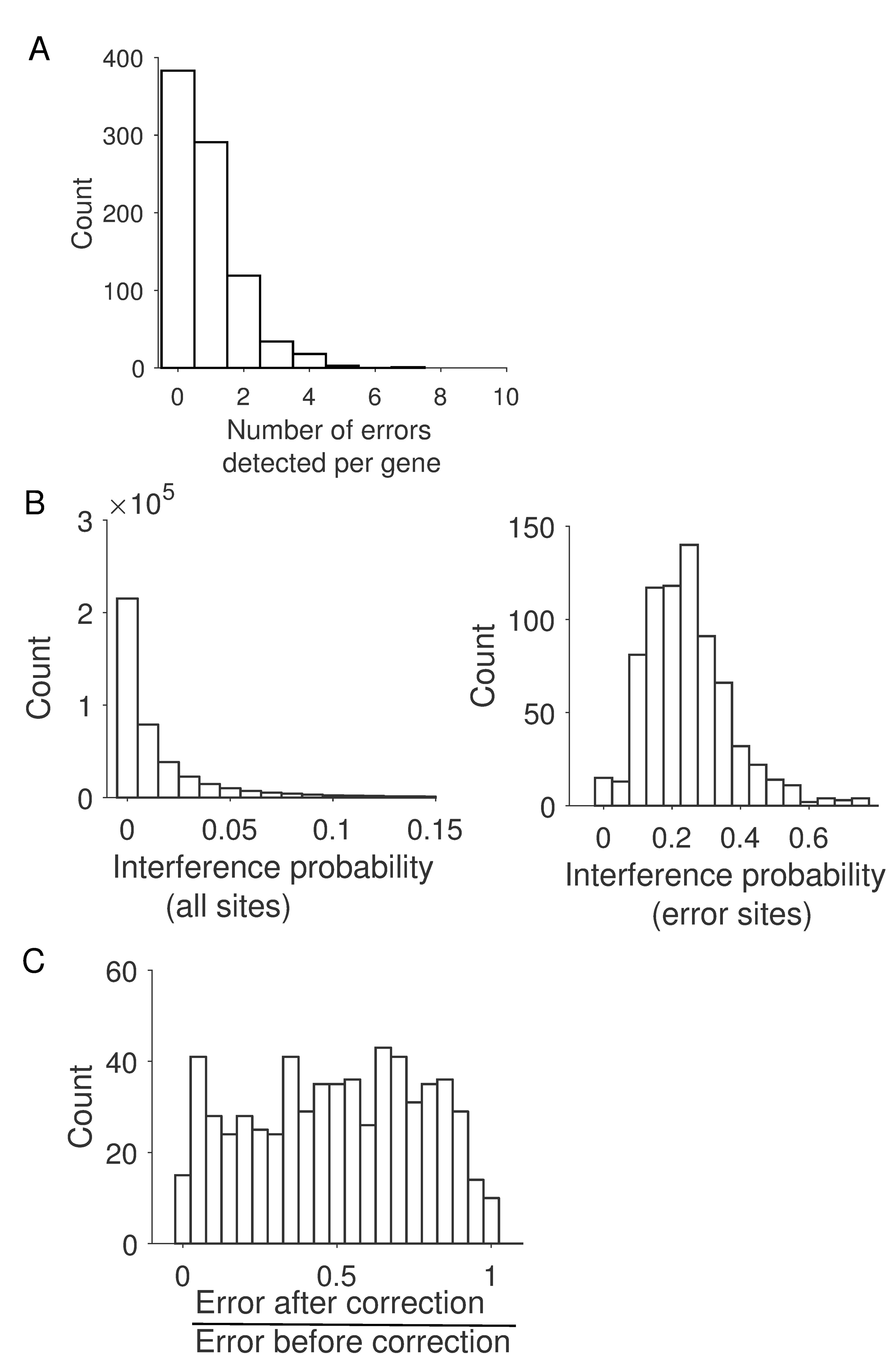

Supplement: S2 Fig — A. Histogram of the number of significant errors detected for each gene. During the inference procedure, 383 genes over the 850 in the dataset (45%) did not require corrections, which means that the difference of profile observed between the simulation and the experiment was for these genes globally under the threshold error fixed by our procedure. For the remaining 467 genes, the number of error sites per gene above the threshold error was on average 1.57 (std = 0.925) B. Histogram of interference probability for significant error sites (left) and for all sites (right). We numerically estimated the probability of a ribosome occupying a certain site to block a ribosome located 10 codons before. We call this empirical probability the interference probability. We found that the interference probability of error sites was on average equal to 0.245 compared with an average rate of 0.011 over all the sites of our dataset. This large difference showed, as we expected, that local profile errors between experimental and simulations after the first round of estimation are primarily due to ribosomal interference. C. Histogram of error improvement after the refinement step, given by the ratio of error after correction over error before. The decrease in error was on average of 57%. For 65% of the sites the error after correction went under the initial threshold of error site detection. The reasons for correction failure can vary from too large initial error, configurations of error sites too close to allow separate correction and more generally possible missing reads or errors in the original data. (TIF) [file pgen.1007166.s003.tif]

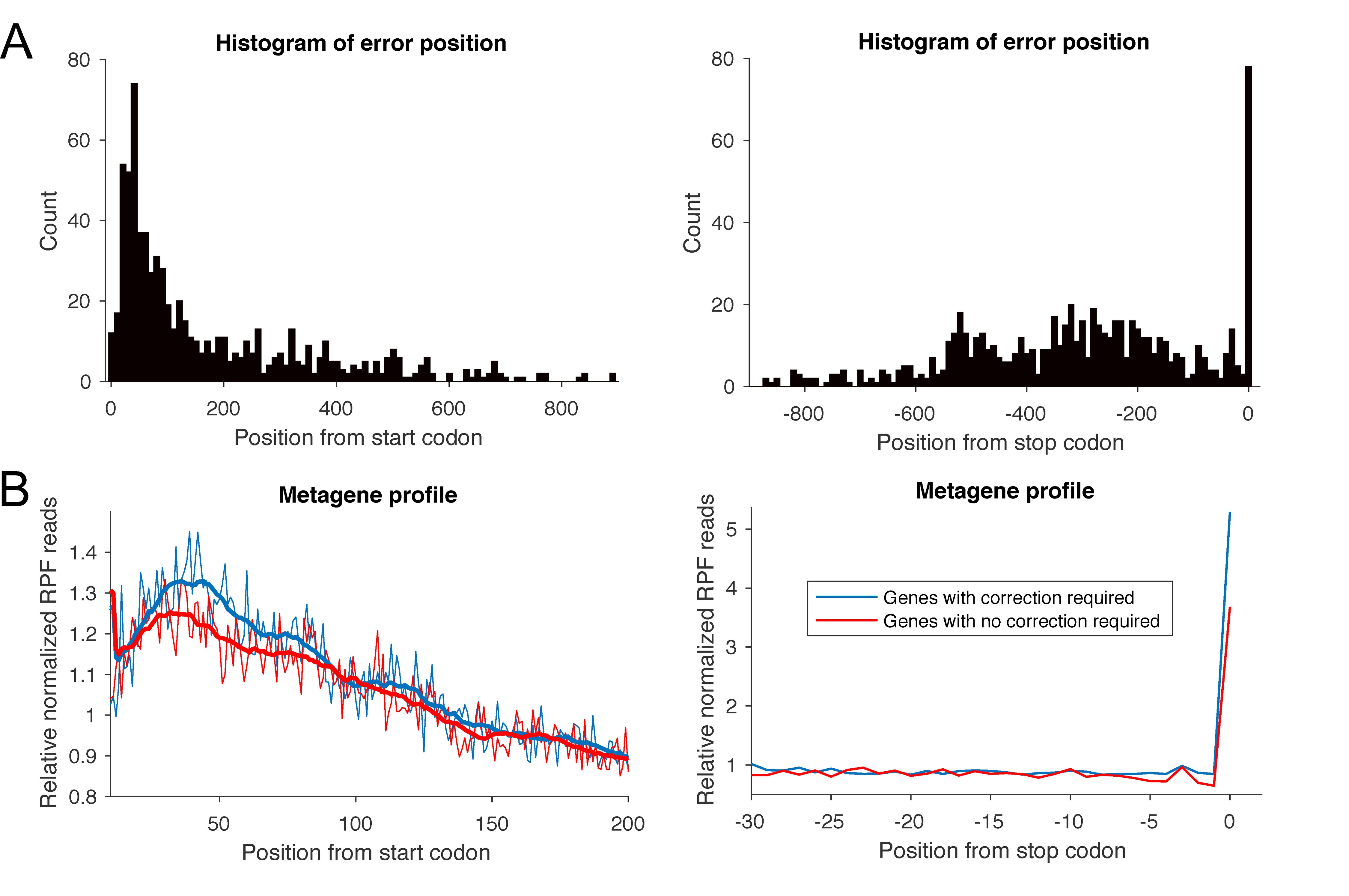

Supplement: S3 Fig — A. Histograms of inconsistent sites obtained from running our procedure on our main dataset, after alignment from start (left) and end (right) position. B. Comparison of metagene profiles (as in Ingolia et al [1]), after alignment from start (left) and end (right) position, between subset of genes that required correction procedure and genes that did not. (TIF) [file pgen.1007166.s004.tif]

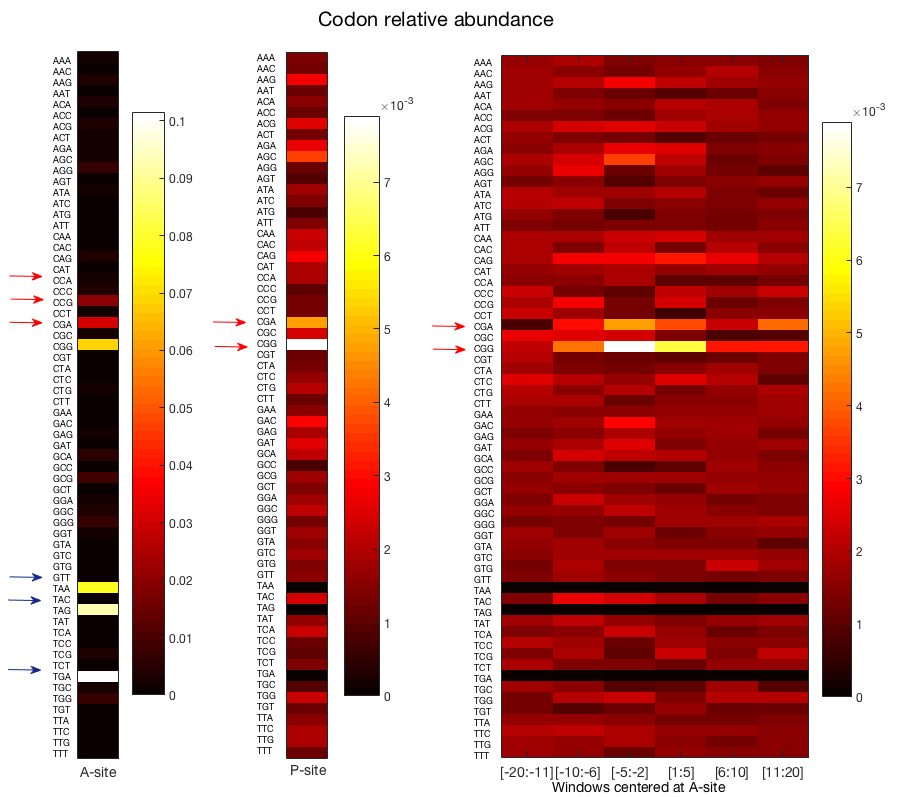

Supplement: S4 Fig — The heatmaps show the relative abundance of each codon (ratio of the local frequency of the codon and its frequency over all the sites) at the A-site, P-site, and other windows ([-20:-11], [-10:-6], [-5:-2], [1:5], [6:10], [11:20]) at inconsistent sites. Blue arrows indicate stop codons. Red arrows indicate non stop codons with slowest mean elongation rate (see Fig 3). (TIF) [file pgen.1007166.s005.tif]

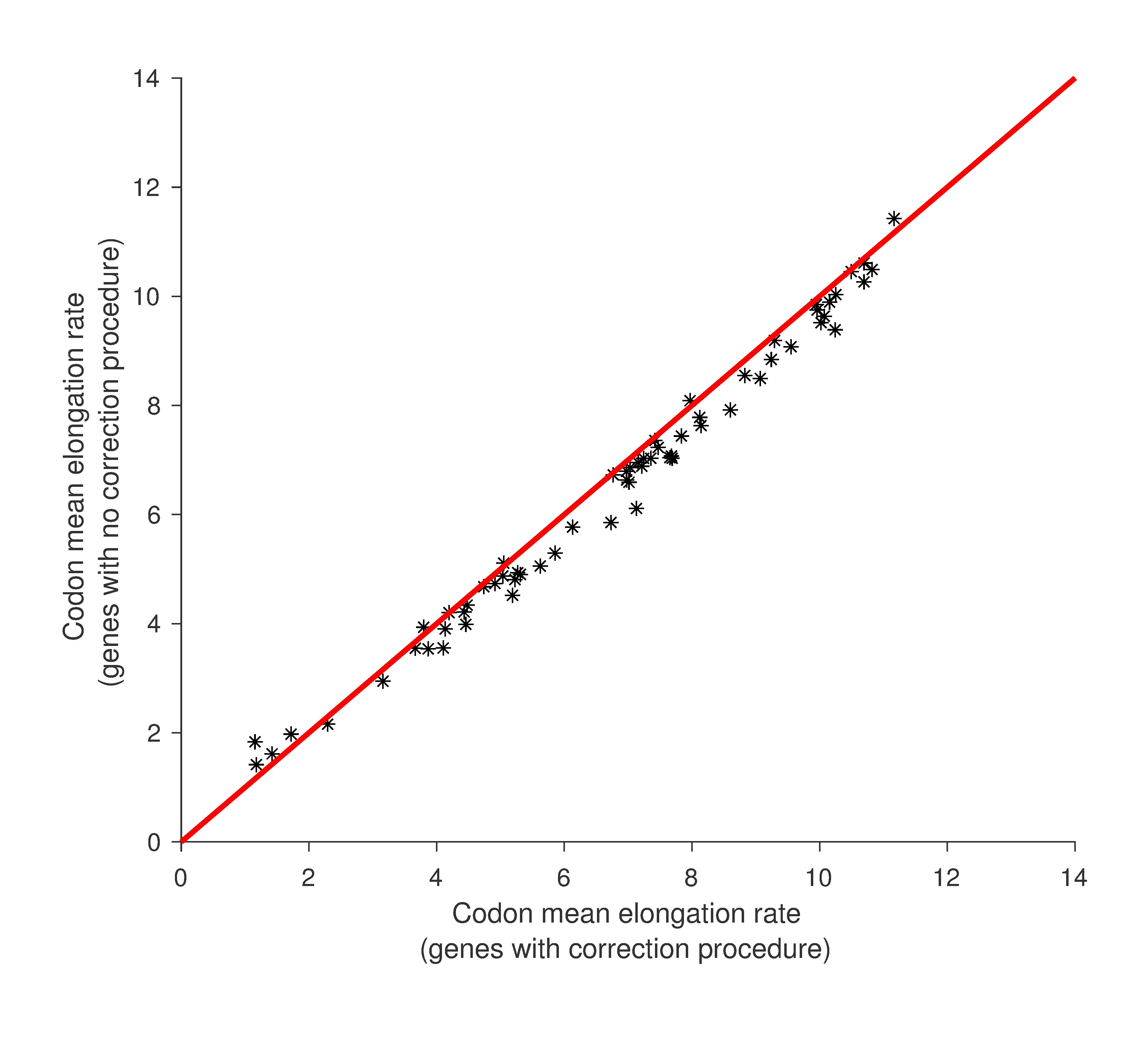

Supplement: S5 Fig — (TIF) [file pgen.1007166.s006.tif]

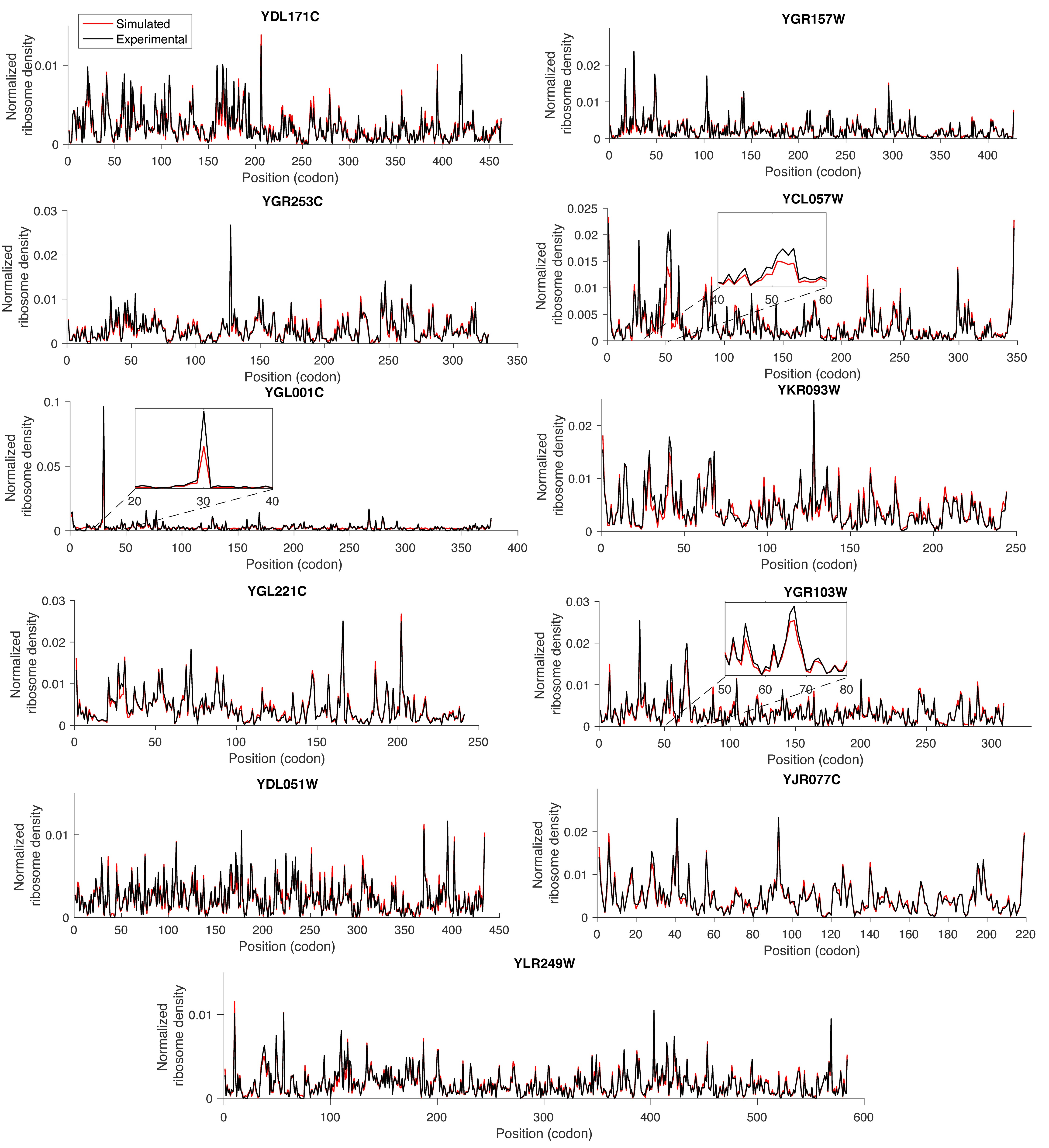

Supplement: S6 Fig — The profiles are obtained by normalizing the ribosome density by the total number of reads. The inset panels (for genes YGR103W, YCL057W and YGL001C) show where the simulated model cannot reproduce the experimental profile. This notably happens when the average observed density is large enough so it gets incompatible with the presence of large peaks. In this case, the model cannot simulate a large peak density without having a queue which leads to stalled ribosomes, and the simulated density is lower than the observed one. (TIF) [file pgen.1007166.s007.tif]

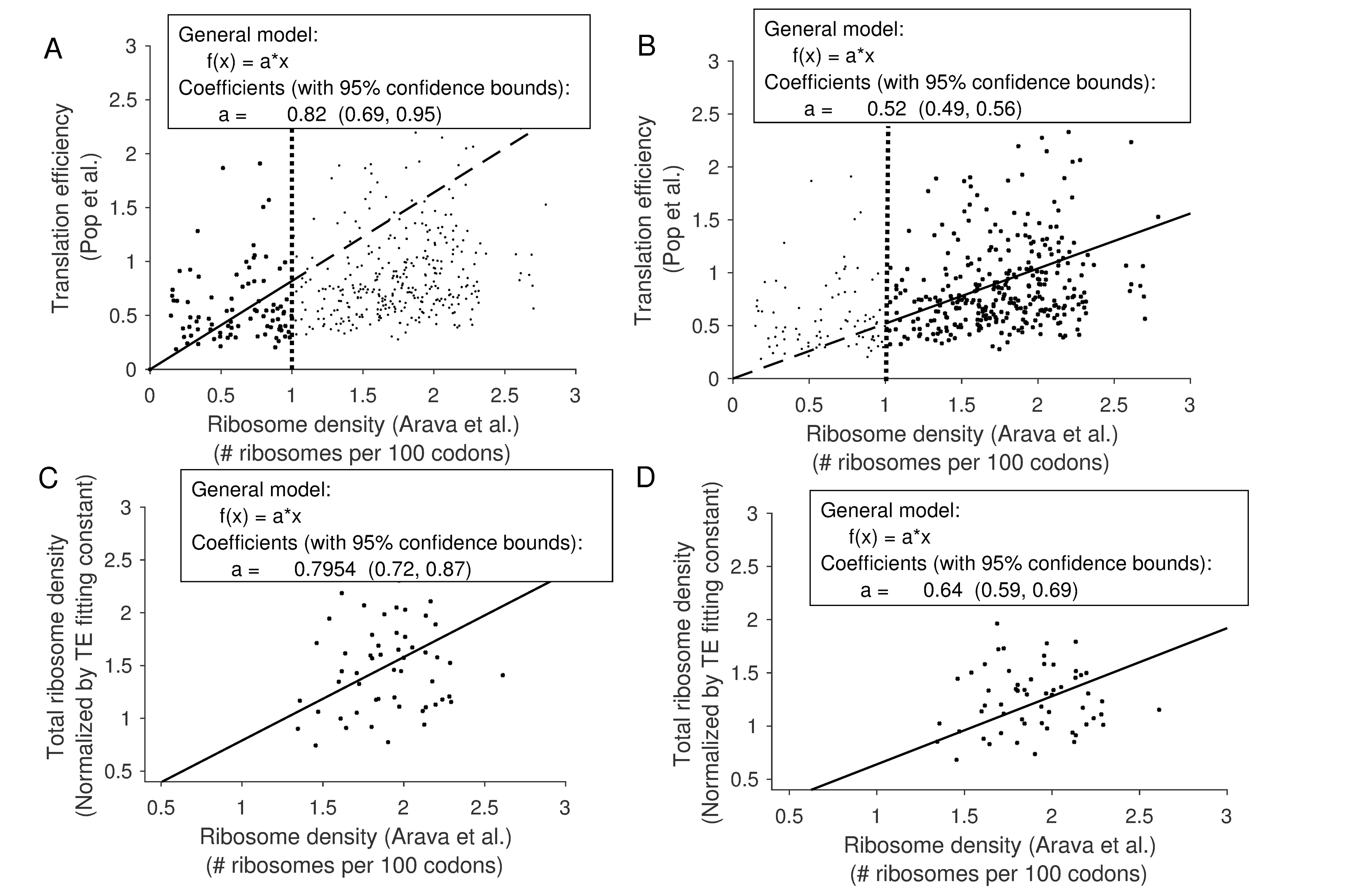

Supplement: S7 Fig — Results from the linear fits are shown in inset. A. The gene-specific TE for 423 genes from Pop et al. [19] (see Materials and Methods) is plotted against the corresponding total ribosome density (average number of ribosomes per 100 codons) from Arava et al. [33]. We performed a linear fit of the points for which the corresponding ribosome density was less than 1 ribosome per 100 codons. B. Similar fit as in A in the range of ribosome density larger than 1 ribosome per 100 codons. C. The simulated total densities for a subset of 58 genes is plotted against the ribosome density from Arava et al.D. The simulated detected-ribosome densities for the same 58 genes is plotted against the ribosome density from Arava et al. (TIF) [file pgen.1007166.s008.tif]

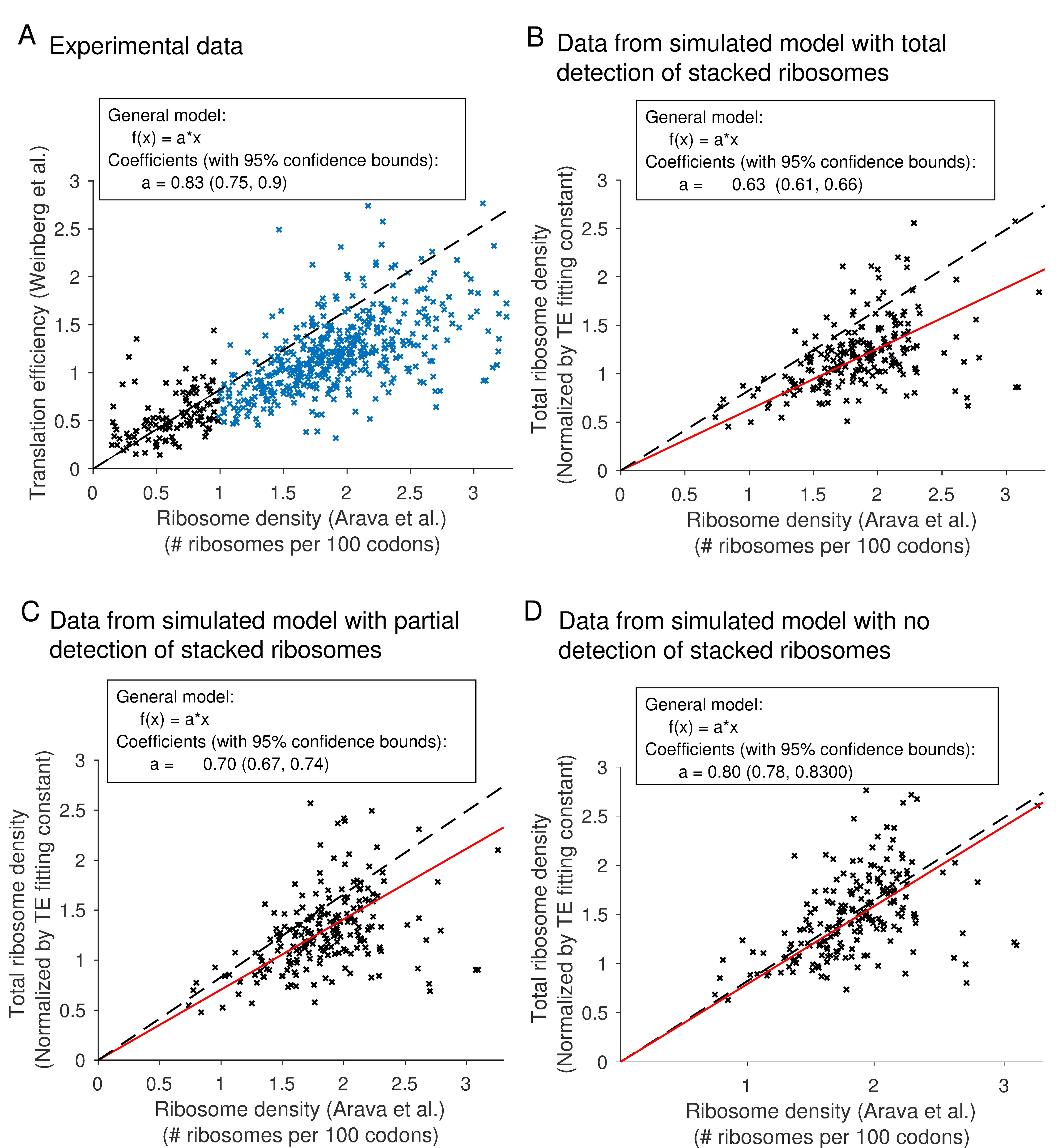

Supplement: S8 Fig — A. We plot the experimental measurement of translation efficiency (ratio of ribosome RPKM and mRNA RPKM) and fit the TE to the density in the region where the density is less than 1 ribosome per 100 codons (linear coefficient 0.83). B. We compare the experimental density to the simulated TE under a model where all ribosomes get detected. The linear fit (plotted in red) between the simulated TE and the density gives a coefficient of 0.63. C. The same as in B, with a model where stacked ribosomes are partially detected with probability 0.5. Linear fit coefficient is 0.70. D. The same as in B, with no detection of stacked ribosomes. Linear fit coefficient is 0.80, that is the best matching with experimental data. (TIF) [file pgen.1007166.s009.tif]

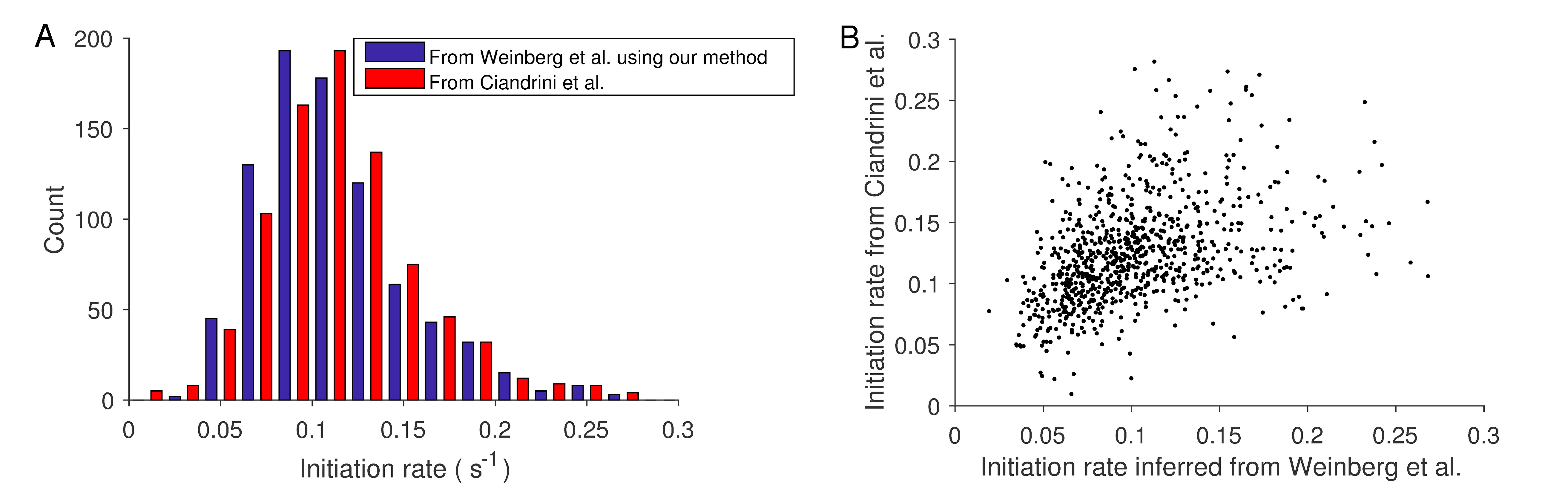

Supplement: S9 Fig — A. Histogram of the two sets of inferred initiation rates. B. Comparison between initiation rates inferred by the two methods. Pearson R = 0.4, p-value< 10−5. (TIF) [file pgen.1007166.s010.tif]

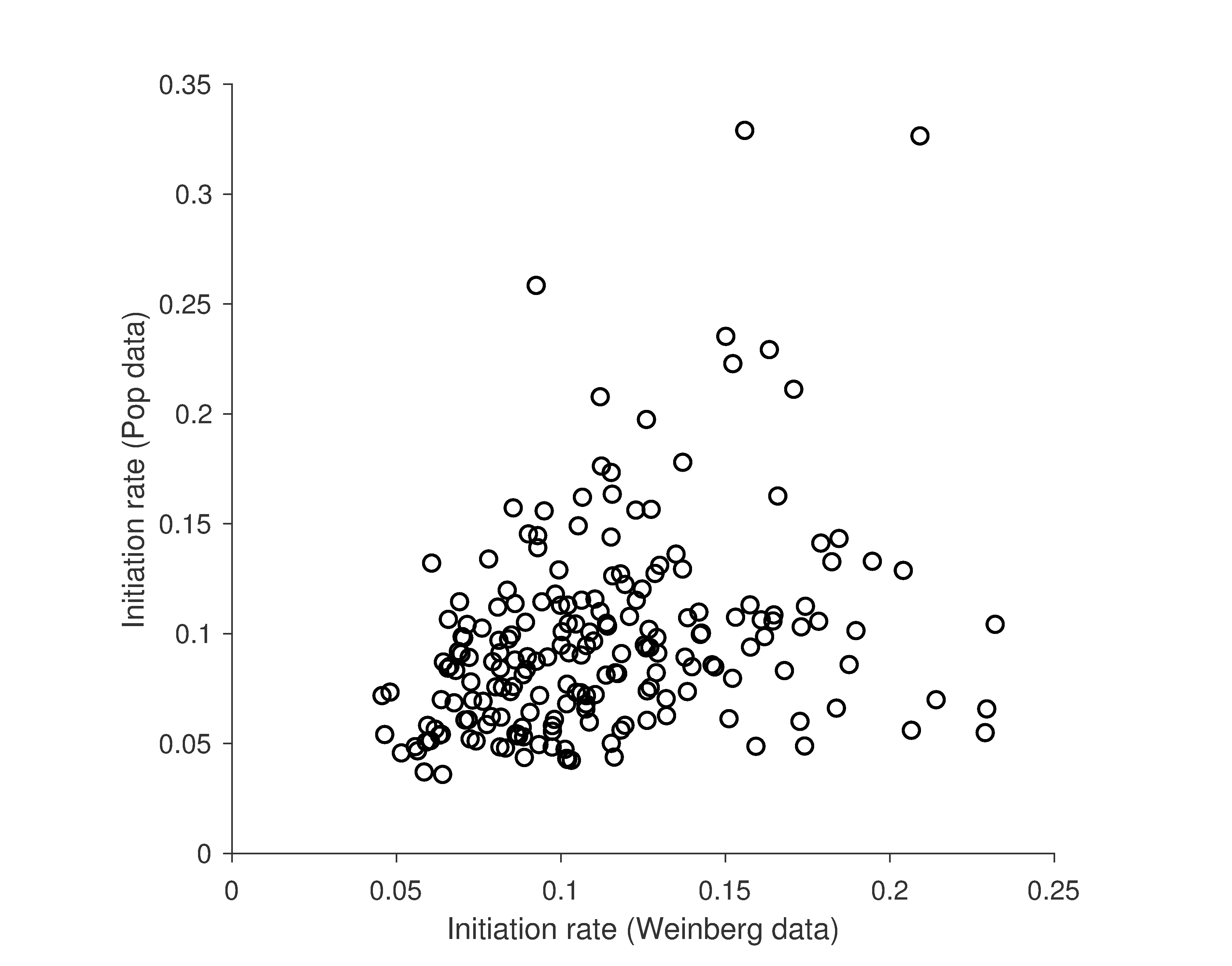

Supplement: S10 Fig — Pearson R = 0.31, p-value< 10−5. (TIF) [file pgen.1007166.s011.tif]

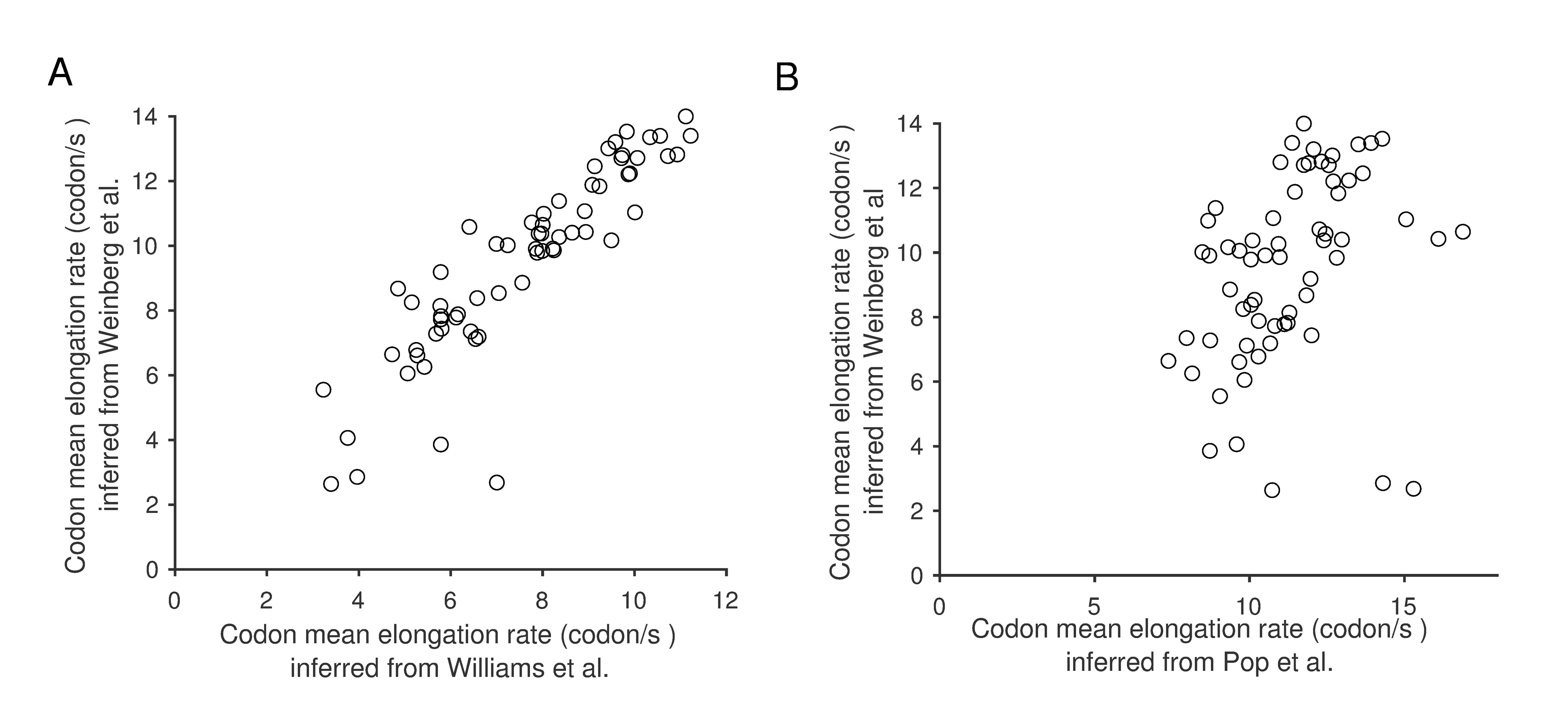

Supplement: S11 Fig — A. We compare the codon-specific mean elongation rates inferred from the Weinberg et al. dataset [16] to the ones inferred from Williams et al. dataset [32]. B Comparison between the codon-specific mean elongation rates inferred from the Weinberg et al. dataset [16] and the ones inferred from Pop et al. dataset [19]. (TIF) [file pgen.1007166.s012.tif]

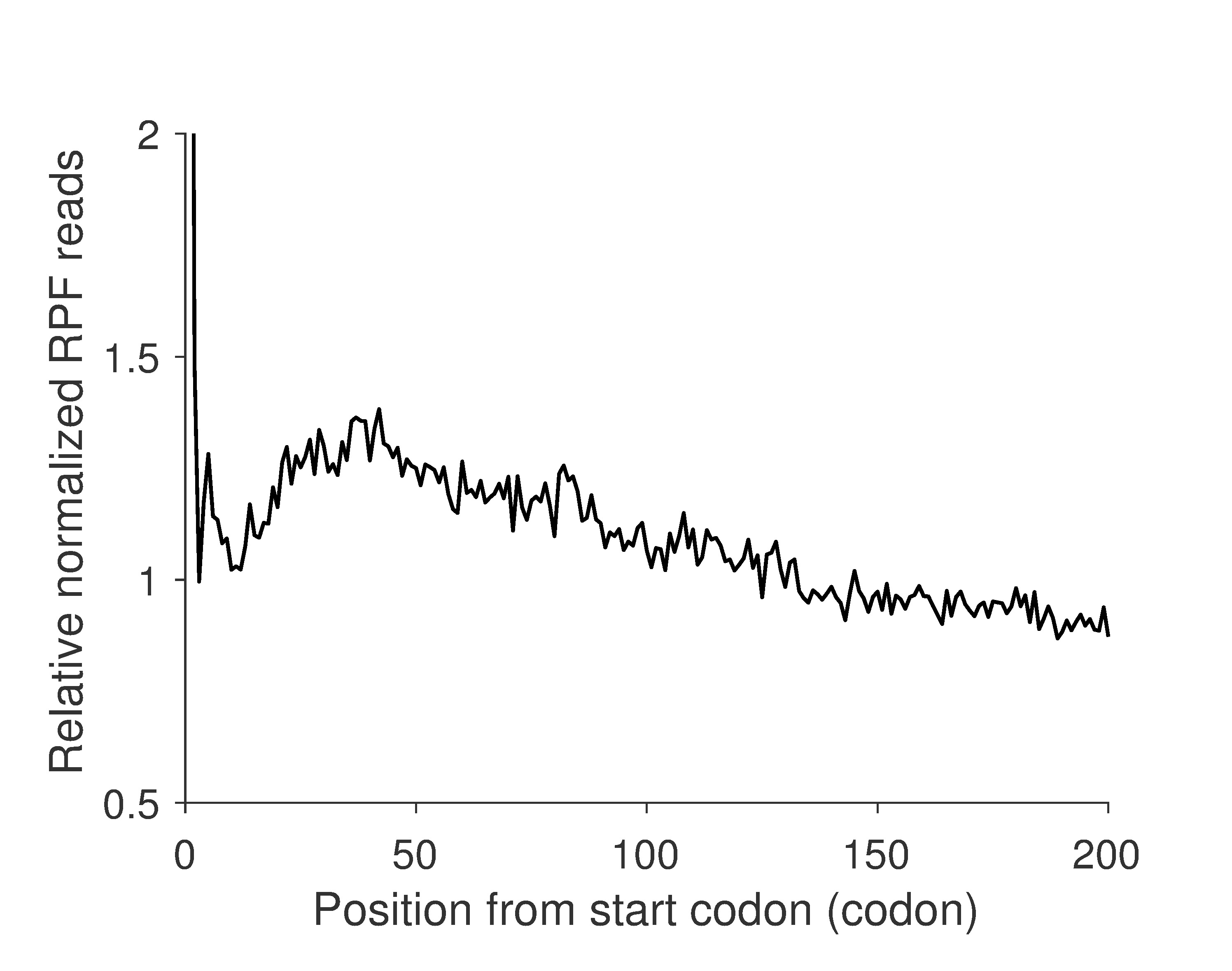

Supplement: S12 Fig — Ribosome profile footprint (RPF) reads in open reading frames (ORFs) from Weinberg et al. [16] were individually normalized by the mean RPF reads within the ORF, aligned from start codon and then averaged with equal weight for each codon position across all ORFs, as in Ingolia et al. [1]. (TIF) [file pgen.1007166.s013.tif]

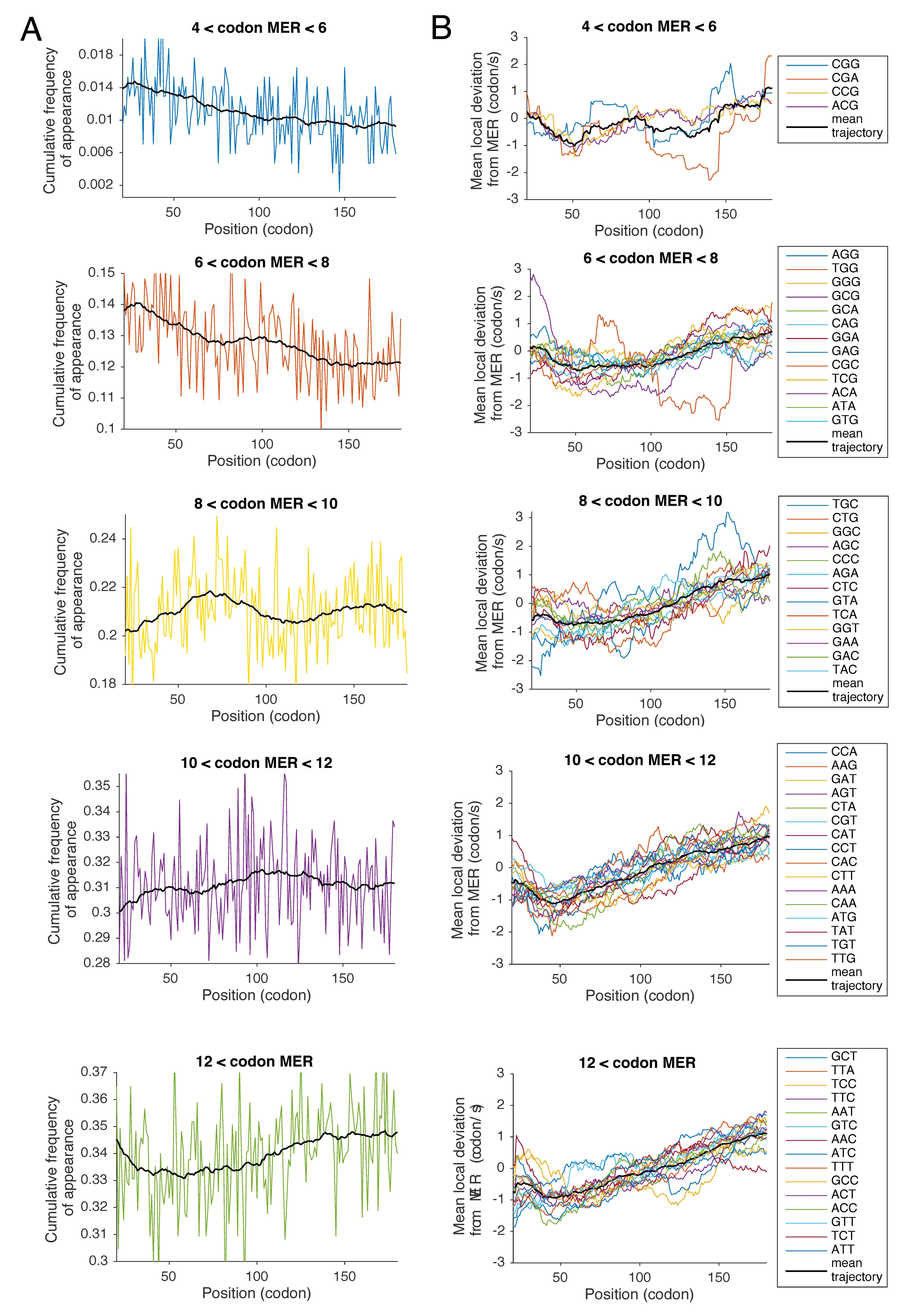

Supplement: S13 Fig — A. Different panels show the frequency of appearance for each group of codons from Fig 5A. The black curve in each panel corresponds to a smoothed version, for which the value at position i is obtained by averaging the values between positions i − 20 and i + 20. B. The difference between codon-specific local speed shown in Fig 5B and the average of codon-specific speeds between position 20 and 180. Different codons are grouped as in A. For each panel, the black curve corresponds to an average of the curves in that panel. (TIF) [file pgen.1007166.s014.tif]

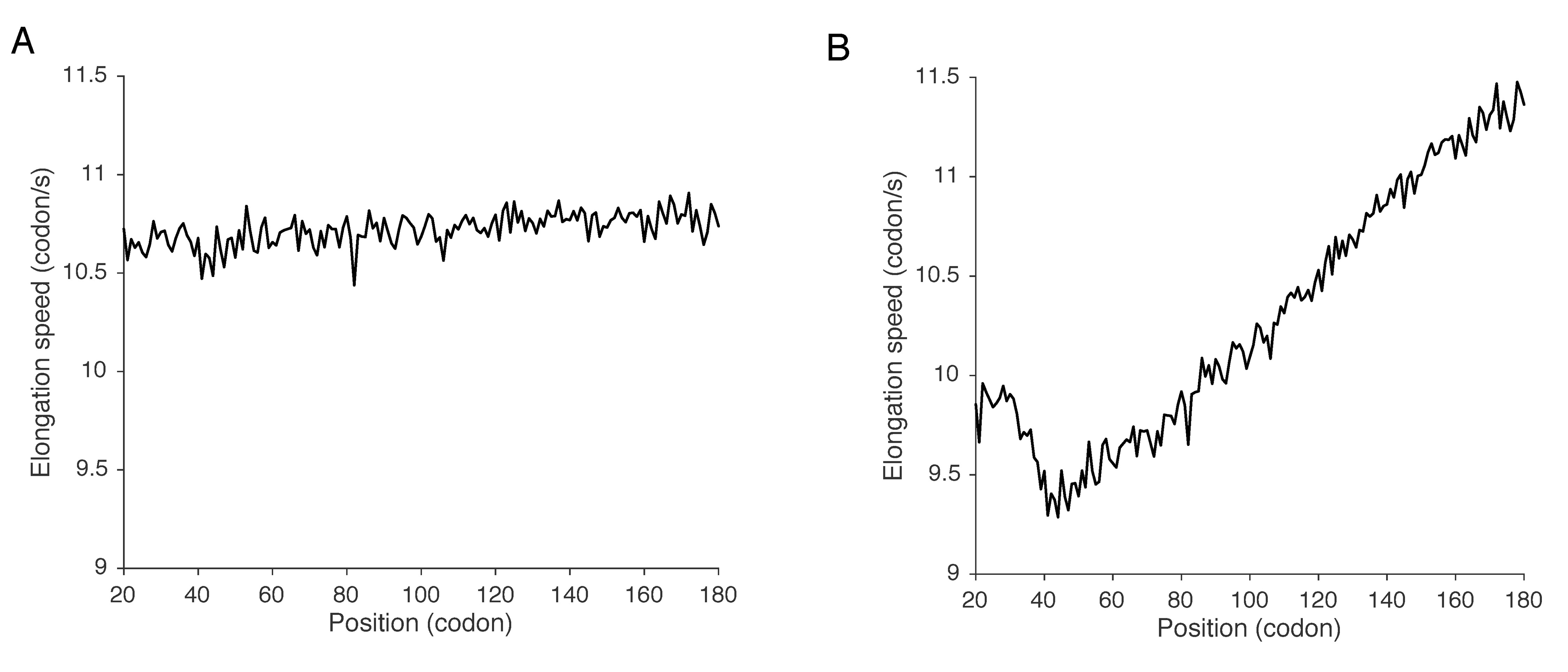

Supplement: S14 Fig — A. Average elongation speed along the transcript obtained by setting the elongation speed for each codon type at all positions to the corresponding mean elongation speed computed from Fig 3B. This plot shows that the variation of codon frequency along the transcript is not sufficient to explain the 5′ translational ramp. B. Average elongation speed along the transcript obtained by setting the elongation speed for each codon to the position-specific mean elongation rate in Fig 5B. (TIF) [file pgen.1007166.s015.tif]

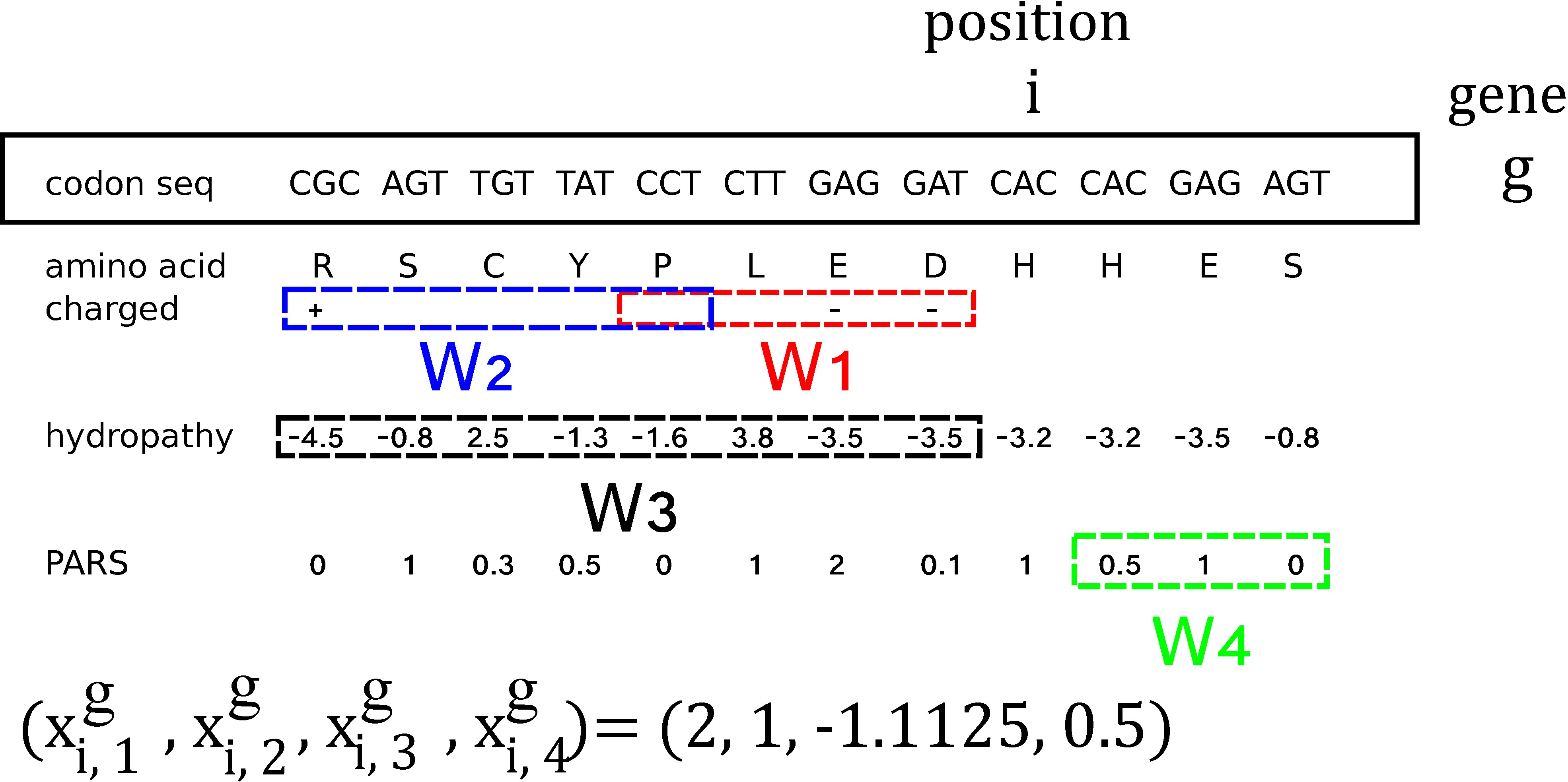

Supplement: S15 Fig — At position i, we consider 4 types of variables in our fitting models: xi,1 is associated with the negative charges, xi,2 with the positive ones, xi,3 with the hydropathy score and xi,4 with the PARS score. For each variable xi,1, a window Wk is defined, such that xi,k=〈xi,kg〉g∈G, where G is the gene set, and for a gene g, xi,kg is computed by averaging (for hydropathy and PARS score) or counting (for the number of charges) the associated feature over positions i − Wk for the charges and hydropathy scores, and positions i + Wk for the PARS score. In this example, W1 = [1 : 4], W2 = [4 : 8], W3 = [1 : 8] and W4 = [1 : 3]. (TIF) [file pgen.1007166.s016.tif]

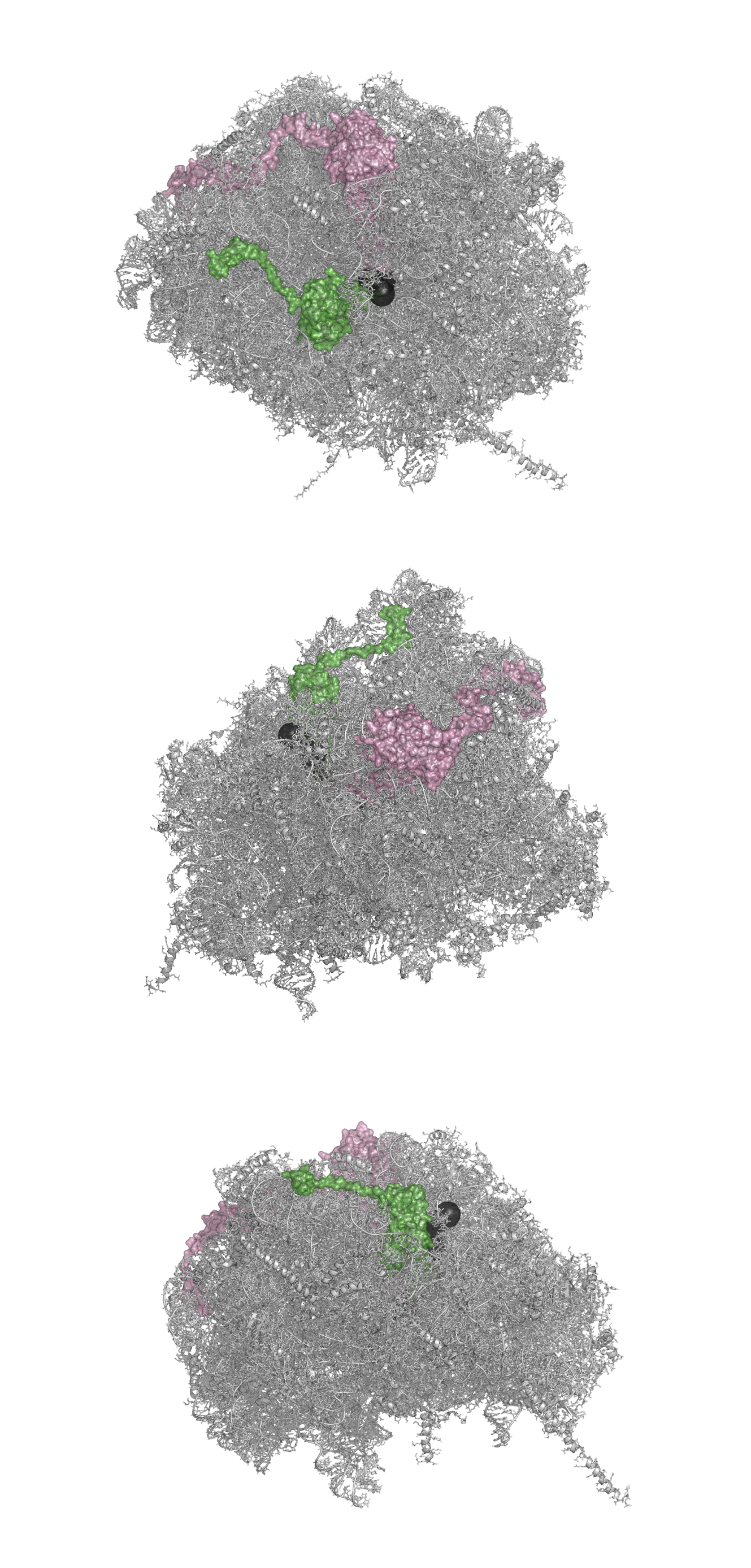

Supplement: S16 Fig — (TIF) [file pgen.1007166.s017.tif]

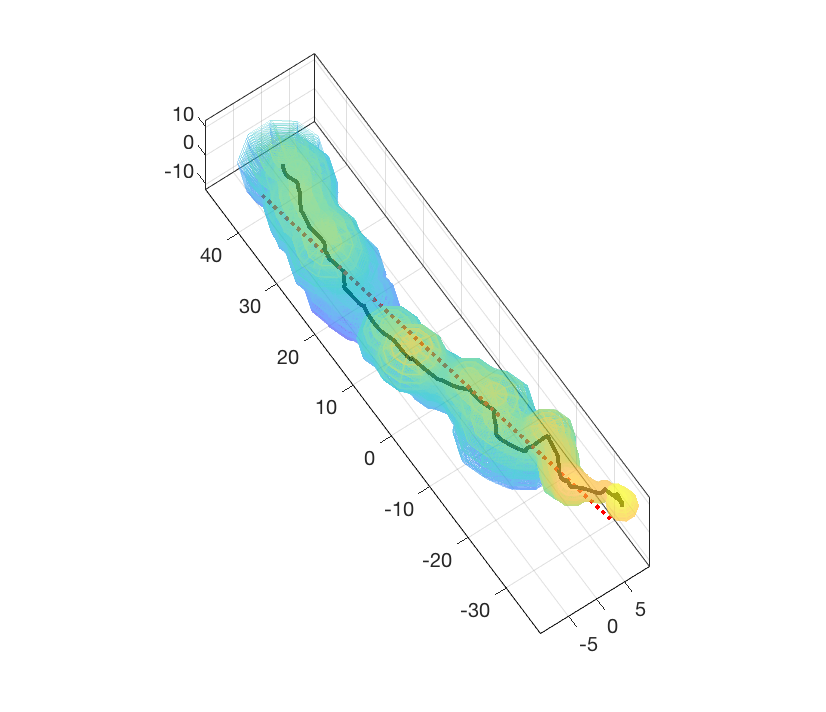

Supplement: S17 Fig — We plot (in black) the centerline of the tunnel (see Material and Methods) from the PTC (bottom right) to the exit (top left)). A linear fit of the centerline (coefficient of determination R2 = 0.985) is shown in red. (TIF) [file pgen.1007166.s018.tif]

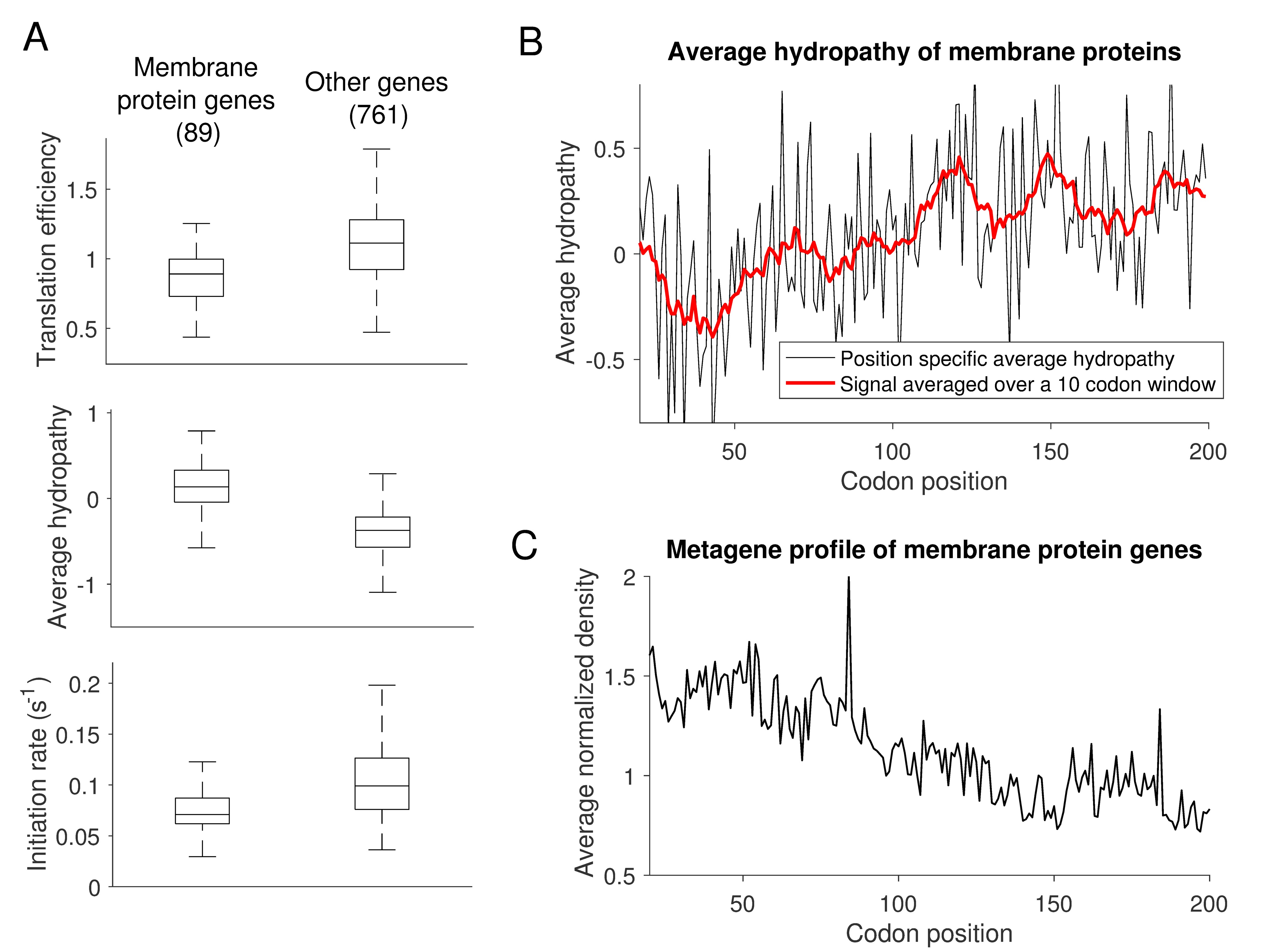

Supplement: S18 Fig — A. We compare the translation efficiency, average hydropathy and the inferred initiation rates of a subset of 89 membrane protein genes to the other genes of our main dataset. These 89 genes were obtained by cross-referencing our main list of genes (850 genes) to a list of 666 genes associated with membrane proteins (from Miller et. al. [55], table 2). We found that the membrane protein genes have in average lower TE (0.88 ± 0.20 compared to 1.10 ± 0.26), larger hydropathy score (0.14 ± 0.28 compared to −0.42 ± 0.3) and lower initiation rates (0.07 ± 0.02 s−1 compared to 0.11 ± 0.05 s−1). Boxplots give the lower and upper adjacent values, first and third quartile and median values. B. The average hydropathy score across codon positions for membrane protein genes. We plot (in black) along the codon position the hydropathy scores averaged over all 89 genes. In red, we plot this average score smoothed by averaging over a 10 codons window (see also S19 Fig). C. Metagene relative normalized ribosome-footprint density as a function of codon position for the 89 membrane protein genes (see also S12 Fig). (TIF) [file pgen.1007166.s019.tif]

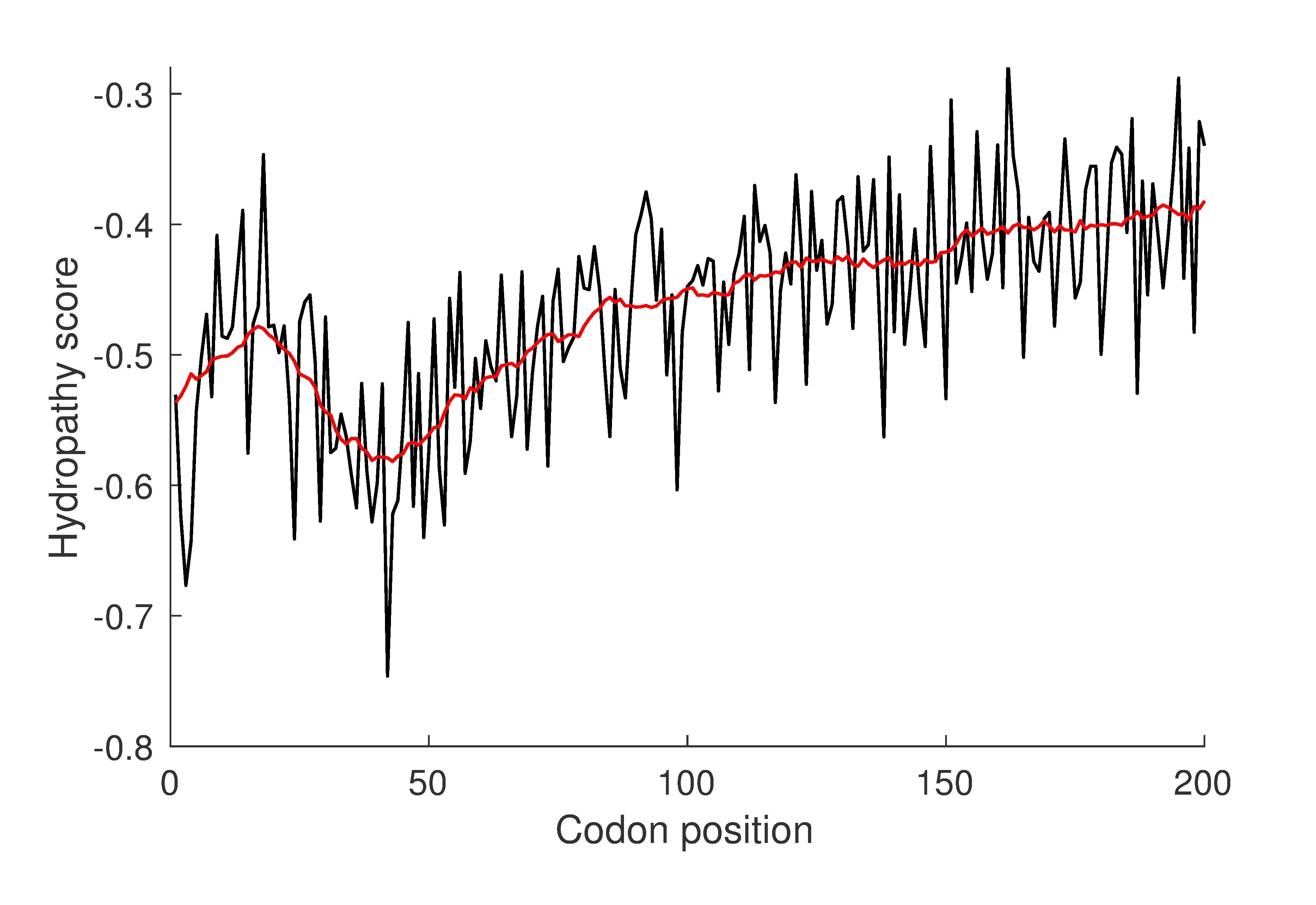

Supplement: S19 Fig — We plot (in black) along the codon position the hydropathy scores averaged over all genes of the non-filtered dataset (2862 genes). In red, we plot this average score smoothed by averaging over a 10 codons window. (TIF) [file pgen.1007166.s020.tif]

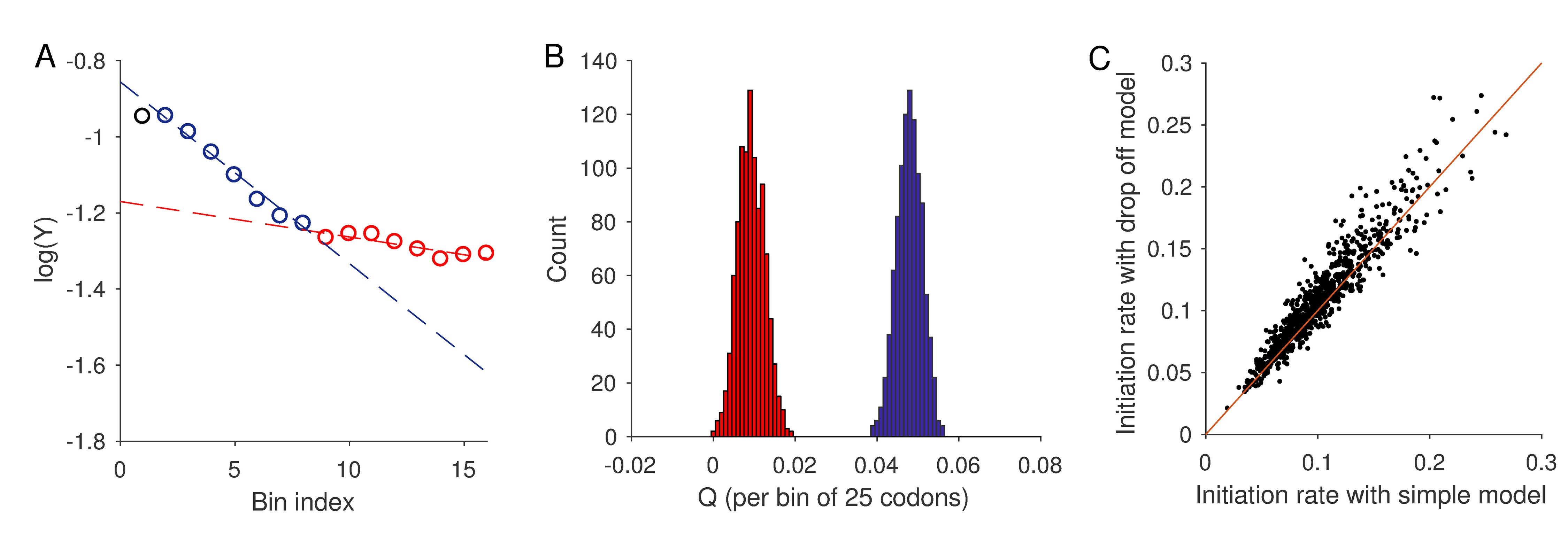

Supplement: S20 Fig — A. We divide the ORF in bins of 25 codons and plot for each bin the logarithm of the average normalized number of reads counted from Weinberg et al. dataset [16]. The associated drop off rate is estimated by linearly fitting the average value of each column with Ae−QX, where X is the bin index and A, Q are fitting parameters. We distinguish two regions of constant drop off rates in windows [25, 225] (in blue) and [200, 400] (in red). Linear fitting (dotted lines) gave Q = 0.048, with resulting drop-off probability per codon and per elongation event r = 0.002) for region [25, 225] and Q = 0.0093 (r = 3.7.10−4) for region [200, 400]. Genes were chosen to be of length > 400 codons and such that the number of reads per codon is on average larger than 10 (458 genes). B. Histogram of Q following the bootstrap method from Sin et al. [22] (number of samples 103). C. We compare the original estimates of initiation rates with the ones obtained with a modified model of translation including drop-off with probability 3.7 × 10^4^ (Pearson R = 0.94, p-value <10−5). (TIF) [file pgen.1007166.s021.tif]

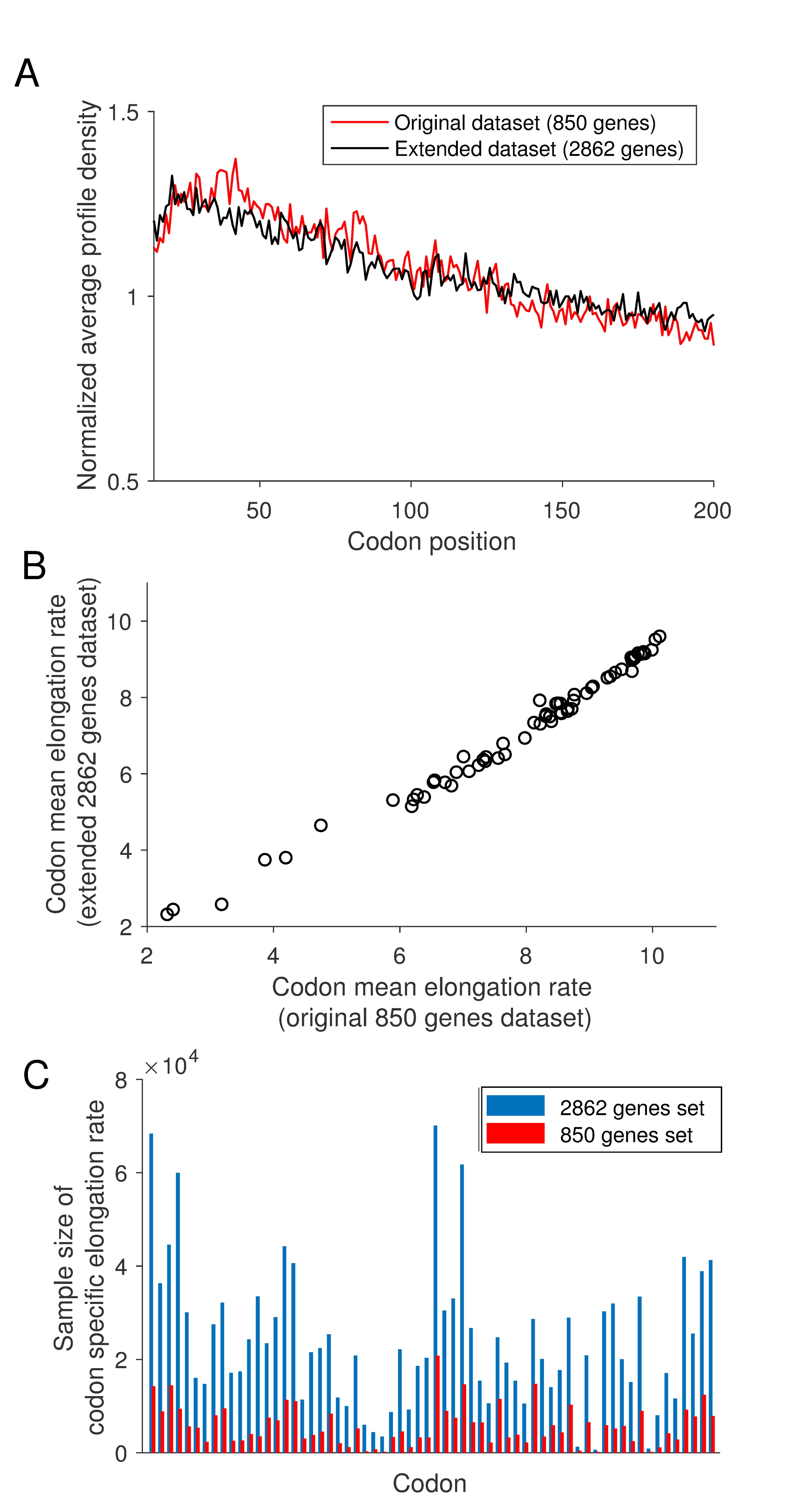

Supplement: S21 Fig — A. We plot (in black) the normalized average profile density across the first 200 codons (as done in S12 Fig) from the extended dataset of 2862 genes (selected to be of length > 200 codons). We compare these variations to the ones obtained (in red) from the filtered dataset used in our main analysis (length > 200 codons and average number of footprints > 10 per codon). Both show the same 5′ “translational ramp” pattern. To obtain the variations for the extended dataset at each position i, the genes whose profile at i contained at least one footprint were considered (for any gene g, pg(i) > 0). These profile values were averaged across the genes, after normalizing each of them by ∑k∈Ig pg(k), where Ig is the set of all positions k where pg(k) > 0 (such processing was done to discard the gene-specific positions that contains no information on the elongation rate variations). B. Comparison between the mean codon elongation rate from the filtered and non-filtered dataset. The elongation rates were obtained using the naive estimates of our inference procedure (see Supplementary section) and filtered by selecting rates < 20 codon/s. This filtering was done to discard the estimates from positions with no or a few footprints, as these estimates are not informative of the dynamics (the threshold of 20 codon/s still allows to keep more than 85% of the inferred rates, for the codon with largest mean elongation rate). C. We plot for each codon and each dataset the sample size of the associated elongation rates used to get the mean codon elongation rate in B. This comparison shows that after filtering for rates <20 codon/s, the extended dataset of 2862 genes still contains for each codon on average ∼5.3 more rates than the original dataset of 850 genes. (TIF) [file pgen.1007166.s022.tif]
